# An Orthogonal Way of Writing the Tubulin Code

**DOI:** 10.1002/cm.70002

**Published:** 2025-06-28

**Authors:** Vicente Jose Planelles‐Herrero, Emmanuel Derivery

**Affiliations:** ^1^ Cell Biology Division MRC Laboratory of Molecular Biology Cambridge UK

## Abstract

The Elongator complex has long been characterized for its role in tRNA modification and modulation of protein translation. Beyond these established functions, recent findings reveal an unexpected role for Elongator in directly interacting and tuning microtubule dynamics and properties, broadening its biological significance in eukaryotic cells. This places Elongator among a growing number of RNA‐binding proteins, such EIF4A3, that have been shown to modulate the microtubule cytoskeleton.

Elongator is a conserved multi‐subunit complex composed of six proteins, Elp1‐6, organized into two stable sub‐complexes, Elp123 and Elp456 (Abbassi et al. 2024; Dauden et al. 2017; Glatt et al. 2012; Jaciuk et al. 2023). Canonically, Elongator modifies wobble uridines at position 34 (U_34_) of specific tRNAs, regulating translation fidelity and efficiency (Dauden et al. 2019; Huang et al. 2005, 2008; Lin et al. 2019; Abbassi et al. 2020). Structural work has captured key intermediates in this process (Abbassi et al. 2024; Jaciuk et al. 2023). Importantly, the two Elongator sub‐complexes participate in distinct stages of the modification process: Elp123 initially binds and modifies the tRNA through its radical S‐adenosylmethionine (rSAM) and lysine acetyltransferase (KAT) domains, after which Elp456 facilitates the release of the modified tRNA, probably in an ATP‐dependent manner (Glatt et al. 2012; Jaciuk et al. 2023).

Beyond its established role in tRNA modification, recent studies have uncovered a moonlighting function of Elongator during cell division, a phase in which translation is largely repressed (Fan and Penman 1970; Stonyte et al. 2018). In a first study, we showed that Elongator controls microtubule density at the central spindle in asymmetrically dividing sensory organ precursor (SOP) cells during *Drosophila* development (Planelles‐Herrero et al. 2022). While Elongator was initially thought to mediate the acetylation of target proteins, including tubulin (Gardiner et al. 2007), subsequent studies have revealed that Elongator controls tubulin acetylation levels indirectly, via the control of protein translation (Even et al. 2021; Shilian et al. 2022). Notably, we found that Elongator's activity on modulating spindle microtubule dynamics is independent of its enzymatic activities and tRNA‐modifying functions. Instead, in vitro assays showed that Elongator binds directly to microtubules, promoting polymerization and stabilizing them by decreasing the catastrophe frequency. This places Elongator among a growing number of RNA‐binding proteins, such EIF4A3, that have been shown to modulate the microtubule cytoskeleton (Alsina et al. 2024).

A striking feature of Elongator's function during asymmetric cell division is its polarized activity. Rather than acting uniformly, Elongator seems to stabilize microtubules on one side of the anaphase spindle. This localized activity is essential for symmetry breaking of the central spindle, which in turn controls the correct segregation of cell fate determinants during SOP division, and thus for proper fly development (Derivery et al. 2015). Perturbing the subcellular localization of Elongator using nanobodies leads to stabilization of the wrong microtubule population, which in turn leads to fate determinants segregating to the wrong cell (Planelles‐Herrero et al. 2022). These findings highlight the importance of the spatial regulation of cytoskeleton regulators: although tubulin levels are likely comparable between daughter cells, local modulation of microtubule stability by polarized factors like Elongator can induce symmetry breaking of the microtubule cytoskeleton.

How Elongator stabilizes microtubules at the molecular level, however, remained unclear. In a second study, we uncovered an unexpected *division of labour* between Elongator sub‐complexes (Planelles‐Herrero et al. 2025). Specifically, Elp123 and Elp456 exhibit distinct and specific binding preferences: Elp123 associates exclusively with polymerized microtubules, whereas Elp456 binds to αβ‐tubulin heterodimers but not to microtubules. Notably, Elp123 localizes specifically to the GTP‐end of dynamic microtubules, both plus‐ and minus‐ends in their growing phase, but this does not alter the dynamics or stability of the microtubules. Thus, unlike other proteins that regulate microtubule dynamics by binding to microtubule ends and modulating lattice parameters (Atherton et al. 2019; Zhang et al. 2018), Elp123 requires additional steps to stabilize microtubules. Instead, Elp123 was found to recruit Elp456 to microtubule ends, which itself brings along αβ‐tubulin heterodimers. We thus propose that Elongator acts as a tubulin shuttle, increasing the local concentration of tubulin at the growing microtubule ends, promoting polymerization and stability. This model is supported by in vitro assays showing that Elongator lowers the critical tubulin concentration required for microtubule polymerization. The mechanism of tubulin release at the growing end remains unknown but may mirror the modular interplay between Elp123 and Elp456 used in tRNA modification (Abbassi et al. 2024; Gaik et al. 2022; Jaciuk et al. 2023). Importantly, however, the binding of Elp456 to tubulin is ATP‐independent, in contrast to the ATP‐dependent binding to tRNA (Abbassi et al. 2024; Glatt et al. 2012).

Most unexpectedly, we found that Elp456 can discriminate between tubulin post‐translational modifications (PTMs). Tubulin is heavily post‐translationally modified in cells (Janke and Magiera 2020). One key modification is polyglutamylation, which is canonically added by enzymes of the TTLL family and modulates the activity of molecular motors and of severing proteins (Bodakuntla et al. 2021; Genova et al. 2023; Lacroix et al. 2010; Lessard et al. 2019; Valenstein and Roll‐Mecak 2016), particularly at the spindle. As TTLL enzymes act on microtubules rather than free tubulin dimers (Kumar and Flavin 1981; Regnard et al. 1998; Shida et al. 2010), the prevailing model is that polyglutamylation primarily alters the behaviour of pre‐existing, long‐lived microtubules. Our findings now offer a way for highly dynamic microtubules to be also polyglutamylated. Indeed, while Elp123 binds indiscriminately to microtubules, Elp456 shows a strong preference for binding to polyglutamylated αβ‐tubulin heterodimers over other forms. As Elp456‐bound tubulin is subsequently incorporated at microtubule ends, this selective binding results in Elongator‐assembled microtubules to be specifically enriched in polyglutamylated tubulin. We confirmed this prediction using in vitro reconstitution assays combined with PTM‐specific antibodies to show that polyglutamylated tubulin is quantitatively incorporated into microtubules during polymerisation by Elongator. Importantly, we demonstrated that this mechanism was functionally relevant in cultured cells but also in vivo in tissues, as reduced Elongator levels cause a decrease in polyglutamylated tubulin at the mitotic spindle.

This finding helps resolve a conceptual paradox. If TTLL enzymes act on pre‐existing stable microtubules, then how are the highly dynamic microtubules, such as the ones at the mitotic spindle, modified? Notably, we observed a ~5‐fold increase in polyglutamylation in metaphase microtubules compared to interphase microtubules (Planelles‐Herrero et al. 2025). Elongator now provides a plausible solution: instead of modifying microtubules post‐assembly, it selectively incorporates pre‐modified tubulin into growing microtubules. Crucially, this mechanism relies on the presence of a pre‐existing pool of polyglutamylated tubulin, as Elongator itself lacks enzymatic activity to generate PTMs *de novo*. Thus, Elongator rather seems to amplify and spatially concentrate pre‐existing modifications.

What controls Elongator's functional switch between translation and cytoskeletal roles? Our in vitro reconstitutions showed that Elp123 cannot bind tRNA while bound to microtubules, suggesting mutual exclusivity between the two activities. Importantly, all our cell‐based and in vivo studies indicate that Elongator acts on microtubules specifically during cell division. One possible explanation is that the translational shutdown accompanying mitosis suppresses its tRNA‐modifying role, freeing it for microtubule regulation. While this idea aligns with the current data, further research is required to determine the nature of such mechanism.

Finally, it is worth noting that Elongator possesses all the key properties allowing for the local control of the microtubule PTM landscape in polarized cells. Indeed, its localization and activity are modulated by polarity cues (Planelles‐Herrero et al. 2022), which could allow it to enrich polyglutamylated tubulin locally. Such a mechanism could help explain the highly non‐uniform PTM distribution in polarized cells. A prime example is neurons, where Elongator is essential for normal development and function (Close et al. 2006; Creppe et al. 2009; Rork et al. 2002). Consistently, mutations in Elongator underlie several neuropathologies (Cheishvili et al. 2011; Close et al. 2006; Slaugenhaupt and Gusella 2002), suggesting that both its canonical and non‐canonical activities may be important in maintaining neuronal integrity. Whether Elongator can control the microtubule cytoskeleton dynamics and polyglutamylation levels in interphase, however, remains to be elucidated.

## Conflicts of Interest

The authors declare no conflicts of interest.

## Data Availability

Data sharing not applicable to this article as no datasets were generated or analyzed during the current study.
